# Supergene validation: A model-based protocol for assessing the accuracy of non-model-based supergene methods

**DOI:** 10.1016/j.mex.2019.09.025

**Published:** 2019-09-24

**Authors:** Richard H. Adams, Todd A. Castoe

**Affiliations:** Department of Biology, The University of Texas at Arlington, Arlington, TX, 76019, USA

**Keywords:** Supergene validation protocol, Bioinformatics, Phylogenetic inference, Phylogenomics, Species trees, Concatenation, Coalescent, Supergene

## Abstract

Genome-scale species tree inference is largely restricted to heuristic approaches that use estimated gene trees to reconstruct species-level relationships. Central to these heuristic species tree methods is the assumption that the gene trees are estimated without error. To increase the accuracy of input gene trees used to infer species trees, several techniques have recently been developed for constructing longer “supergenes” that represent sets of loci inferred to share the same genealogical history. While these supergene methods are designed to increase the amount of data for gene tree estimation by concatenating several loci into “supergenes” to increase gene tree accuracy, no formal protocols have been proposed to validate this key “supergene” concatenation step. In a recent study, we developed several supergene validation strategies for assessing the accuracy of a popular supergene method: the so-called “statistical binning” pipeline. In this article, we describe a more generalizable and model-based “supergene validation” protocol for assessing the accuracy of supergenes and supergene methods using model-based tests of phylogenetic congruency.

• Supergenes are validated by adopting model-based tests of topological congruence
• These model-based procedures out preform non-model based methods for supergene construction
• The results of this protocol can be used to assess the overall performance of a supergene method across a phylogenomic dataset

## Graphical abstract

**Figure:**
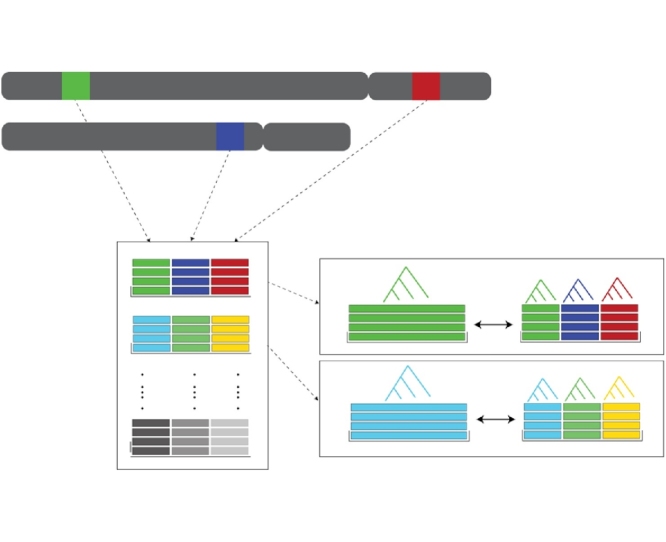

**Specification Table**

**Table:** 

Subject Area:	*Biochemistry, Genetics and Molecular Biology*
More specific subject area:	*Molecular phylogenetics; Bioinformatics*
Method name:	*Supergene validation protocol*
Name and reference of original method:	Adams R.H., and Castoe, T.A. 2019. Statistical binning suffers profound model violation due to gene tree error incurred by trying to avoid gene tree error. Accepted at Molecular Phylogenetics and Evolution. *The reference ID number for this original manuscript is***MPE_2018_449.** Link: https://www.sciencedirect.com/science/article/pii/S1055790318305153?via%3Dihub
Resource availability:	*NA*

## Method details

### Background

Species tree inference is the primary objective of most phylogenetic studies, and while genomic data hold great promise for resolving species-level relationships with high resolution, deriving meaningful inferences from such large and complex datasets is rarely – if ever – straightforward. To address many of the computational challenges presented by large phylogenomic datasets, a number of heuristic approaches have recently been developed to increase the scalability and performance of the species tree algorithm. These approaches typically implement a two-part procedure whereby individual genealogical trees are first estimated for each genomic locus using maximum likelihood (ML) analyses, and the resulting gene tree estimates are used as input to reconstruct a species tree under the multispecies coalescent model using programs such as MPEST [1], ASRAL [2], ASTRID [3], or STEM [4]. This two-part procedure of heuristic methods effectively represents a “divide and conquer” strategy that treats gene tree and species tree estimation as distinct statistical problems.

At the core of heuristic species tree approaches is the assumption that the input ML gene trees are estimated without error. This fundamental assumption has serious implications for most empirical phylogenomic studies for which ML gene trees are often inferred from relatively short loci (i.e., < 10 kb), and thus, are likely to suffer high error rates [5,6]. To increase the accuracy of input gene trees, a third step is often applied to construct longer “supergenes” by concatenating sets of loci that show evidence of sharing the same genealogical tree (i.e., congruent) – these concatenated loci are then treated as a single, recombination-free “supergene” locus. There are a number of “supergene methods” that can be used to infer whether two or more loci share the same topology, and if so, concatenate these loci to construct a single representative supergene (for example [[7], [8], [9], [10]]:). Currently, however, few supergene methods are readily scalable for inferring supergenes from large, genome-scale datasets. First designed to facilitate the avian phylogenomics project [11], the “statistical binning” pipeline [11] is one popular supergene approach that is feasible for genomic analyses because it utilizes its own heuristic threshold to evaluate whether non-model based measures of tree support genealogical congruence (or not). More specifically, statistical binning uses nonparametric bootstrap support (BS) values to assess whether two trees are compatible at a level that is above or below a chosen threshold. If the BS values at all conflicting nodes in both trees are below the threshold, the loci are deemed to be congruent with one another, and thus, are concatenated to form a supergene. Conversely, a supergene is not constructed from two or more loci that have at least one conflicting node with a support value higher than the threshold.

Regardless of which supergene method is used, the inference of genealogical congruence among loci for the purposes of supergene construction is a fundamentally different statistical problem than either gene or species tree inference that is concerned with the combinability of different loci. For non-model based approaches, such as statistical binning, it may be particularly important to understand the statistical properties of the method because downstream phylogenetic inference of the supergene trees is typically conducted using standard model-based phylogenetic frameworks, such as ML analysis or Bayesian inference (BI), which make a number of explicit assumptions about the phylogenetic process. Importantly, ML-analysis of a concatenated supergene assumes that the supergene itself is comprised only of loci that do, indeed, share the same overall tree. In other words, the inferred supergene is assumed to be constructed without error; this is because the standard phylogenetic likelihood function as implemented in the vast majority of model-based phylogenetic inference frameworks assumes that all sites within an alignment evolved along the same tree [12]. When ML-analysis is attempted on a “false supergene” (i.e., supergene that includes two or more discordant loci), the resulting ML tree cannot be accurate because the loci used to infer it do not share the same tree (i.e., only a single tree is inferred when there should be multiple). Recently, the validity of statistical binning and similar methods has been called into question based on both empirical and theoretical work suggesting that these methods can be unreliable when the input gene trees suffer from high estimation error [[13], [14], [15], [16]].

**Fig. 1:**
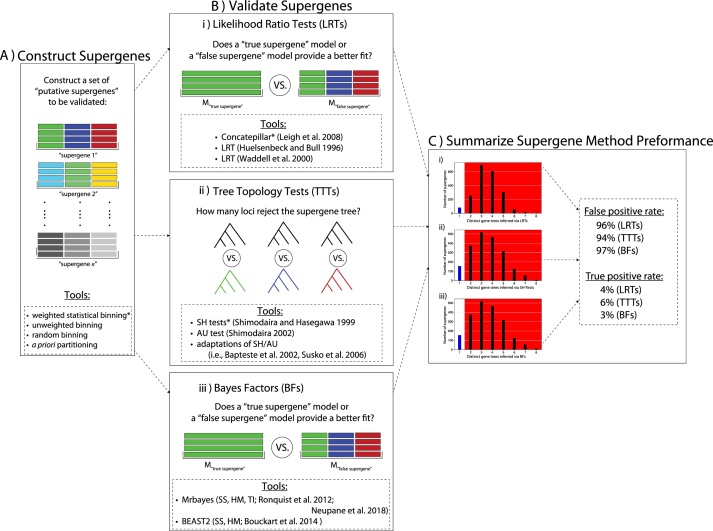
General framework for the supergene validation protocol as applied in Ref. [15]. First, a set of supergenes is obtained (a) either using an a priori concatenation strategy (i.e., partitioning loci into supergenes assumed to share a common tree), or using a heuristic supergene method (such as statistical binning). Here a supergene is depicted as a set of loci (colored alignments) that have been concatenated together to form a single alignment. After a set of supergenes has been constructed, we show three approaches (b) and associated example toolsets that can be used to assess supergene validity (i) Likelihood Ratio Tests (LRTs), (ii) Tree Topology Tests (TTTs), and (iii) Bayes Factor (BF) model comparison. For the LRTs, the fit of two alternative competing models (“true” vs “false” supergene models) is compared, and the best fit model indicates the reliability of the supergene (top box). A number of TTTs can be used to quantify the number of loci that reject or the overall supergene tree topology (i.e., colored vs gray tree shown in center box). BFs can be used in a similar manner to LRTs to compare the fit of “true” and “false supergene models (bottom box). Finally, the results of the supergene validation tests can be used to summarize the overall performance of the supergene method by estimating the number of supergenes that selected the “true” or “false” supergene models – providing an estimate of the false positive and true positive rate of the supergene method, respectively. Asterisks (*) indicate specific analyses used in the original supergene validation study of [15] (Concatepillar and SH tests).

Here we describe a model-based protocol for assessing the validity of supergenes, and therefore, supergene methods, for a given dataset. In a recent study, we applied this “supergene validation” strategy to characterize the poor performance of the statistical binning pipeline for inferring supergenes [15], however, these same principles can foreseeably be used to characterize the performance of nearly any supergene or concatenation method. Data concatenation has been a long-standing practice in the field of systematics, and our protocol can be used prior to phylogenetic tree inference to assess whether concatenated loci are likely to share the same tree (or not). Our general supergene validation protocol is depicted in Fig. 1. While our primary goal is not to review in detail all possible phylogenetic tests that could be used for such a purpose (see [10]), we provide several tools that proved useful for assessing supergene validation in our original study [15], and we also mention additional techniques that could foreseeably be used for supergene validation in a similar manner. Our protocol for supergene validation consists of three main components: (1) a set of supergenes is constructed (Fig. 1a), (2) the validity of each supergene is assessed using (i) likelihood-ratio tests of topological model fit (Fig. 1b, top box), (ii) tree topology tests (Fig. 1b, center box), and/or (iii) Bayes Factor model comparison (Fig. 1b, bottom box), and finally, (3) the results of the supergene validation analyses are summarized to assess the overall performance of the supergene method across an entire phylogenomic dataset (Fig. 1c).

### Constructing a set of supergenes

A set of supergenes can be obtained in a number of ways, but the primary goals are the same: decide which loci share a common tree (and if so, combine into a supergene) and which loci do not share a common tree. For relatively small phylogenetic datasets, *ad hoc* supergenes are often constructed based on *a priori* knowledge of gene features (i.e., codon positions, exons, introns), which forms the basis of partitioned phylogenetic analyses of concatenated data [17]. For example, many phylogenetic studies conduct ML-analysis of concatenated datasets constructed from a handful of nuclear and/or mitochondrial protein coding genes whereby each gene and codon position are assigned an independent nucleotide substitution model, but only a single tree is assumed for all loci [[17], [18], [19], [20]]. For larger phylogenomic analyses, heuristic non-model-based supergene methods (such as statistical binning) are often used to construct supergenes based on evidence of tree congruency among loci.

For the purpose of this article and to provide the same context as our original supergene validation study [15], we primarily discuss the use of our supergene validation protocol for assessing the accuracy of the statistical binning method that was used to infer supergenes for the avian phylogenomic analyses [11,[21], [22], [23]]. We refer readers to the original studies [21,23] describing the statistical binning technique in detail, which generally involves the inference of individual gene trees and bootstrap support values that are used to build an incompatibility graph, which itself is used to infer whether individual loci can be concatenated together to form supergenes (or not). In any case, the processes of supergene inference (whether conducted *a priori* or with a more formal supergene method; Fig. 1a) defines the process of partitioning a multilocus dataset into a non-overlapping set of supergenes.

### Supergene validation

Central to the purpose of supergene validation is assessing whether an inferred supergene is statistically justified using model-based topological congruency tests (Fig. 1b). In other words, after a supergene (or set of supergenes) has been inferred (either based on *a priori* assumptions or via a more formal supergene method; Fig. 1a), the goal is to test whether the individual loci placed within a supergene should be treated as a single concatenated locus with a single phylogenetic tree topology (i.e., “true supergene”) or not (i.e., “false supergene”). There are a number of techniques designed for testing phylogenetic congruency (for a recent review, see [10]), and our supergene validating protocol seeks to leverage these techniques for the propose of validating both inferred supergenes themselves, and in turn, the overall supergene method (i.e., Fig. 1c). We discuss our supergene validation protocol in light of three primary types of topological congruency tests that can be used for this purpose: (i) Likelihood Ratio Tests (LRTs), (ii) Tree Topology Tests (TTTs), and (iii) Bayes Factor model comparison (BFs). These techniques are discussed below and depicted in Fig. 1b alongside a handful of example tools and software that can be used for this purpose.

(i) Likelihood Ratio Tests
  Phylogenetic tree models can be compared using hierarchical likelihood ratio tests (LRTs, Fig. 1b), which have been applied in a number of contexts for comparing the relative fit of statistical models of molecular evolution to sequence data [24,25], as well as for testing alternative tree hypotheses [9,10]. In the context of supergene validation, we can apply LRTs to test whether the fit of a “true supergene” model ($M_{“\text{true supergene}”}$) containing only a single tree is significantly better than a “false supergene” model ($M_{“\text{false supergene}”}$) that includes multiple distinct trees (Fig. 1b). The likelihood of a supergene alignment D given $M_{“\text{true supergene}”}$ is computed as $L\left(\text{D|}M_{“\text{true supergene}”}\right)$, and similarly, the likelihood given $M_{\text{false supergene}}$ is $\;L\left(\text{D|}M_{“\text{false supergene}”}\right)$. The ratio of these two likelihoods can be computed as follows:
  $$LRT=\frac{L\left(\text{D|}M_{“\text{true supergene}”}\right)}{L\left(\text{D|}M_{“\text{false supergene}”}\right)}$$
  Or more commonly, the ratio of log-transformed likelihoods:
  $$LRT_{log}=\;L_{log}\left(\text{D|}M_{“\text{true supergene}”}\right)-L_{log}\left(\text{D|}M_{“\text{false supergene}”}\right)$$
  with significance determined using an assumed distribution or simulations. Accepting a “true supergene” model suggests that a particular supergene was accurately constructed because the best fit model was that of a single tree for all loci. Conversely, rejecting this “true supergene” model in favor of a “false supergene” model means that a particular supergene is likely a false positive because the best fit model included multiple, distinct topologies for loci that comprise that supergene (Fig. 1b, top box).
  In our original demonstration of supergene validation using the avian phylogenomic analysis [15], we use the LRT framework implemented in the program Concatepillar [26], which conducts a series of hierarchical LRTs to test the total number of distinct trees supported by the inferred supergene. In other words, for each supergene, Concatepillar seeks to identify the best fit model containing anywhere from 1 to *n* trees, where *n* is the number of distinct loci placed within a supergene. If the best fit model indicated by the LRT includes only a single tree (i.e., n =1), then supergene is likely a true positive, while evidence for the existence of multiple, distinct gene trees (i.e., n≥1) suggests a false supergene. In addition to Concatepillar, several other software programs and statistical frameworks exist for estimating the number of trees for a given alignment, include the LRTs as derived in Huelsenbeck and Bull [27], as well as Waddell et al. [28]. In any case, the goal is to leverage phylogenetic LRTs to assess whether the supergene is statistically justified and validated under the assumptions of the standard phylogenetic model [12].
(ii) Tree Topology Tests
  A number of likelihood-based Tree Topology Tests (TTTs) and statistical frameworks have been developed for comparing the fit of two topologies to a dataset, and several of these approaches can be adopted for conducting topology tests of congruency (Fig. 1b, center box; see [10]). For each inferred supergene (i.e., each supergene in the set shown in Fig. 1a), and for each of the *n* loci placed within each supergene (i.e., colored alignments in Fig. 1a), we use TTTs to quantify the number of loci that reject the overall supergene topology (i.e., gray tree in Fig. 1b, center box) in favor of its own locus-specific distinct gene tree (i.e., colored trees in Fig. 1b, center box). The Shimodaira-Hasegawa (SH) test is a popular useful test for comparing statistical support for two conflicting phylogenetic tree hypotheses [29], and here we adopt the SH test for supergene validation by quantifying the number of loci placed within a supergene that either accept or reject the overall supergene tree (Fig. 1b, center box). Evidence in favor of a true supergene is indicated by a failure of all *n* loci to reject the supergene topology. If one or more loci reject the supergene tree, this suggests the supergene is a false positive. To use the SH for validating supergenes, a set of ML gene tree estimates are needed for each locus within a supergene (i.e., colored trees in Fig. 1b, center box), as well as the overall supergene tree itself (i.e., gray tree shown in Fig. 1b, center box). SH tests are implemented as standard functions in several phylogenetic programs, including RAXML [30], which we used in our original supergene validation study [15]. Additionally, other TTTs (e.g., AU [31];) can likely be used in a similar manner to the SH test for the purpose of supergene validation.
(iii) Bayes Factors
  Bayes Factors (BFs) have received recent attention for their utility in comparing the fit of phylogenetic models within a Bayesian framework using marginal likelihood estimates [32]. Although we did not use BFs in our original study [15], we propose that BFs (Fig. 1b, bottom box) can be used for validating supergenes in a similar manner as to LRTs (Fig. 1b, top box). Indeed, a recent study has shown that BFs can be useful for assessing the degree of combinability among loci [33], and thus, this approach is well-suited for determining whether a supergene is statistically justified (or not). A marginal likelihood $P\left(D,M_{i}\right)$ represents the overall average fit of a statistical model M to a given dataset D [34], and the average model fit of two competing models $M_{i}$ and $M_{j}$ can be compared using BFs as a ratio of marginal likelihoods:
  $$BF=\frac{P\left(M_{i},D\right)}{P\left(M_{j},D\right)}$$
  BFs have been applied in a variety of phylogenetic and evolutionary contexts to compare the fit of nucleotide substitution models [35], demographic models [36,37], and molecular clock models [38], to name a few. Similar to the example use of LRTs for validating supergenes (Fig. 1b, top box), we can use BFs to compare the fit of a “true supergene” and “false supergene” model as follows:
  $$BF=\frac{P\left(M_{“\text{true supergene}”},D\right)}{P\left(M_{“\text{false supergene}”},D\right)}$$
  where $P\left(M_{“\text{true supergene}”},D\right)$ is the marginal likelihood for the “true supergene” model in which all loci in the supergene alignment D share the same tree (gray alignment in Fig. 1b, bottom box), and $P\left(M_{“\text{false supergene}”},D\right)$ is the marginal likelihood of a “false supergene” model in which each locus has its own separate gene tree (colored alignments in Fig. 1b, bottom box). In this context, BFs provide an intuitive measure of how much a putative supergene favors a “true supergene” against a “false supergene” model. Analytical computation of marginal likelihoods is intractable for all but the most simple phylogenetic models, and techniques such as Harmonic Mean Estimation (HM [39];), Stepping Stone analysis (SS [40];), and Thermodynamic Integration (TI [41];) are typically used to approximate marginal likelihoods.
  There are a number of Bayesian phylogenetic inference programs, such as BEAST [42] and MrBayes [43] that implement marginal likelihood estimation and thus could be used to estimate the marginal likelihoods for the purpose of supergene validation. Similar to the use of LRTs, BFs in favor of a single-tree model would indicate that the supergene method provided a “true supergene”. However, if BFs support a multi-tree model (2 or more trees), than the supergene is likely to be a false positive, which should not be used for downstream species tree inference.

### Characterizing the statistical performance of a supergene method

We can summarize the results of various supergene validation approaches (i.e., LRTs, TTTs, and/or BFs, Fig. 1b) to characterize the performance of a supergene method (Fig. 1c). This can be accomplished by exploring the supergene validation results both independently and collectively in a number of ways. Each approach has its own advantages and disadvantages, and one can use this information to understand and assess the supergene method according to different validation procedures (Fig. 1b). For example, the total percentage of false supergenes that appear to incorrectly inferred can be quantified using the LRTs, TTTs, or BFs. For LRTs and BFs, the overall performance of the supergene method can be assessed by quantifying the number of supergenes for which the best-fit model was comprised only of a single supergene tree (i.e., Fig. 1b). For example, in the case of the avian phylogenomic analyses, we found that >93% of supergenes inferred via statistical binning appeared to be false supergenes according to LRTs [15]. Similarly, the number of genes that reject the overall supergene tree topology can be quantified across the entire supergene set to understand whether the supergene method was reliable or not using TTTs. The results of the three different supergene validation approaches (i.e., LRTs, SH-tests, and BFs) can be compared to assess their agreement (or lack thereof). In any case, false supergenes can be effectively treated as suspect, and investigated further to understand their biological and evolutionary properties. Importantly, this supergene validation strategy allows the independent assessment of which supergenes adhere to the assumptions that all loci comprising a supergene share a common genealogy, and thus are appropriate for use in downstream species tree inference.

### Conclusions

The practice of concatenating distinct loci together to form supergenes has been applied extensively in phylogenetic analyses for decades as an attempt to increase statistical accuracy of gene tree inference. Despite its widespread application in the field, the statistical justification for using this popular technique is rarely assessed in empirical analyses. This is concerning given recent evidence that many supergene methods exhibit unreliable behavior that may mislead inference [16], and in the context of large genome-scale datasets, this error may compound to more strongly mislead species tree inference. The supergene validation protocol described in this article provides a general and extendable framework for evaluating the validating of supergenes. We expect that this approach will be useful for further investigations into the causes and consequences of phylogenetic conflict while providing the means to obtain more reliable species trees from larger genome-scale data.
