# Supplementary material for: Supergene validation: A model-based protocol for assessing the accuracy of non-model-based supergene methods
Source: MethodsX. 2019 Sep 24;6:2181–8. doi: 10.1016/j.mex.2019.09.025 (PMC6812401; doi:10.1016/j.mex.2019.09.025)

Accepted Manuscript

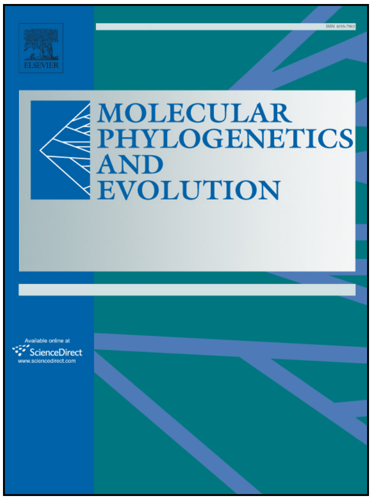

Statistical binning leads to profound model violation due to gene tree error incurred by trying to avoid gene tree error

Richard H. Adams, Todd A. Castoe

PII: S1055-7903(18)30515-3
DOI: https://doi.org/10.1016/j.ympev.2019.02.012
Reference: YMPEV 6426

To appear in: *Molecular Phylogenetics and Evolution*

Received Date: 10 August 2018
Revised Date: 30 November 2018
Accepted Date: 14 February 2019

Please cite this article as: Adams, R.H., Castoe, T.A., Statistical binning leads to profound model violation due to gene tree error incurred by trying to avoid gene tree error, *Molecular Phylogenetics and Evolution* (2019), doi: https://doi.org/10.1016/j.ympev.2019.02.012

This is a PDF file of an unedited manuscript that has been accepted for publication. As a service to our customers we are providing this early version of the manuscript. The manuscript will undergo copyediting, typesetting, and review of the resulting proof before it is published in its final form. Please note that during the production process errors may be discovered which could affect the content, and all legal disclaimers that apply to the journal pertain.

ACCEPTED MANUSCRIPT

Statistical binning leads to profound model violation due to gene tree error incurred by trying to avoid gene tree error

Richard H. Adams[1] and Todd A. Castoe[1,*]

[1] Department of Biology, The University of Texas at Arlington, Arlington, TX, 76019, USA

[*]**Correspondence**: Todd A. Castoe, Department of Biology, University of Texas at Arlington, Arlington, TX 76010 USA

Email: todd.castoe@uta.edu *phone:* 817-272-9084 *fax:* 817-272-9615

Keywords: phylogenetic inference; phylogenomics; species trees; concatenation; coalescent; supergene

ACCEPTED MANUSCRIPT

ACCEPTED MANUSCRIPT

Abstract

Fundamental to all phylogenomic studies is the notion that increasing the amount of data – to entire genomes when possible – will increase the accuracy of phylogenetic inference. Simply adding more data does not, however, guarantee phylogenomic inferences will be more accurate. Even genome-scale reconstructions of species histories can suffer the effects of both incomplete lineage sorting (ILS) and gene tree estimation error (GTEE). Weighted statistical binning was originally proposed as a technique to assist the avian phylogenomics project in solving the bird tree of life, which has long eluded resolution as a result of both ILS and GTEE. These so-called "statistical binning procedures" seek to overcome GTEE by concatenating loci into longer multi-locus "supergenes" that are used to reconstruct a species tree under the assumption that the supergene tree set is an accurate estimate of the true underlying gene tree distribution. Here we evaluate the performance of the method using the original avian phylogenomics dataset. Our results suggest that statistical binning constructs false supergenes that concatenate loci with different coalescent histories more often than not: >92% of supergenes comprise discordant loci. Our results underscore a major logical inconsistency: GTEE – the sole justification for using statistical binning instead of standard concatenation – also makes these methods unreliable. These findings underscore the need for developing new robust frameworks for phylogenomic inference that more appropriately accommodate GTEE and ILS at a genome-wide scale.

ACCEPTED MANUSCRIPT

ACCEPTED MANUSCRIPT

1. Introduction

Much of our understanding and practice of evolutionary biology relies on knowledge of the species-level relationships of organisms (i.e., species trees). Two major sources of phylogenetic conflict can pose serious challenges for species tree reconstruction: incomplete lineage sorting (ILS) and gene tree estimation error (GTEE). Standard phylogenetic analysis of concatenated loci, for example, will be statistically inconsistent in the presence of ILS and yield highly-supported but incorrect species trees (Edwards et al., 2007; Kubatko and Degnan, 2007). To address this, coalescent-based methods have been developed that are statistically consistent under ILS and will return the true species-level phylogeny with high confidence given sufficient information (Degnan and Rosenberg, 2009; Heled and Drummond, 2010; Knowles, 2009; Liu, 2008; Liu et al., 2010, 2015b). While ILS is an inherent property of the demographic processes of speciation and divergence, GTEE is a fundamentally different source of conflict that represents statistical sampling error and variation between the true tree and one estimated from a dataset of finite size and information content. Although modern phylogenomic datasets often consist of millions to billions of base pairs (bp), any one aligned locus is often limited to <3kbp of aligned orthologous sequence data, and thus individual gene trees may entail substantial error that can permeate to the level of species tree inference (Jarvis et al., 2014; Mirarab et al., 2014). Researchers thus face a gauntlet of challenges when analyzing phylogenomic data: concatenate loci and suffer the consequences of ILS, or do not concatenate loci and suffer the consequences of GTEE. Both sources of conflict can have major debilitating effects on the accuracy of species tree estimates, and it is not immediately clear whether one should prioritize either.

The avian Tree of Life is a prime example of an important vertebrate phylogeny that has long eluded resolution because of both ILS and GTEE (Jarvis et al., 2015; Mirarab et al., 2014; Prum

ACCEPTED MANUSCRIPT

ACCEPTED MANUSCRIPT

et al., 2015). In light of the challenges facing phylogenomic analyses, a new method ("weighted statistical binning"; referred to as "statistical binning" hereafter) was originally developed to enable the avian phylogenomics project in resolving the relationships of modern birds (Bayzid et al., 2015; Jarvis et al., 2015; Mirarab et al., 2014). The method has since been used to infer the evolutionary relationships of placental mammals (Tarver et al., 2016), teleost fishes (Malmstrøm et al., 2016), and many other major radiations (i.e.., Blaimer *et al.* 2016; Branstetter *et al.* 2017; JeŠovnik *et al.* 2017; Platt *et al.* 2018). The core justification behind this approach is to infer a set of "supergenes" that attempt to overcome GTEE by concatenating smaller sets of individual loci into longer supergene alignments comprising multiple loci that contain more information for inferring supergene trees. In practice, supergenes inferred via statistical binning are often used to obtain a set of supergene trees for downstream species tree estimation under the assumption that that they are 100% accurate. Importantly, gene tree estimates and associated bootstrap support values are used as input data for the statistical binning pipeline as the sole criteria for deciding whether the respective loci within a putative supergene evolved under the same tree (Bayzid et al., 2015). Using a compatibility graph based on these estimates, the pipeline effectively conducts a hypothesis test to decide whether several individual loci can be concatenated to form a supergene (i.e., they share a common topology) or not (i.e., do not share a topology; Mirarab *et al.* 2014; Bayzid *et al.* 2015). Accordingly, the fundamental purpose of statistical binning is to infer which phylogenetic conflicts among estimated gene trees are simply a result of GTEE (result: concatenate to form a supergene), and which conflicts represent true differences in coalescent history due to ILS (result: do not concatenate and estimate distinct trees).

Following publication of the avian phylogenomics project, substantial debate and contention has arisen over the use of statistical binning and similar methods (Bayzid et al., 2015; Jarvis et al.,

ACCEPTED MANUSCRIPT

2015; Liu and Edwards, 2015; Mirarab et al., 2015; Roch and Warnow, 2015; Warnow, 2015). Authors have continued to argue both for and against these methods, and disagree over the statistical consistency (or lack of) of these approaches in the context of species tree estimation (Liu and Edwards, 2015; Mirarab et al., 2015; Roch and Warnow, 2015; Warnow, 2015). A follow-up study revealed that statistical binning distorted supergene tree distributions and likely biased species tree estimates (Liu and Edwards, 2015). Further studies corroborated this assertion: species trees reconstructed using supergenes obtained via statistical binning were likely to be highly inaccurate yet highly supported (Streicher et al., 2018). Subsequent response papers rejected the assertion that the method was statistically inconsistent, and instead argued for statistically consistency when the number of loci and the length of loci are both infinite (Bayzid et al., 2015; Mirarab et al., 2015). However, recent theoretical work has demonstrated the inconsistency of species tree methods that use supergenes inferred via statistical binning when the number of loci is unbounded but the length of each locus is bounded to a constant (Roch et al., 2018). These findings raise important questions about the nature of species tree inference under best-case scenarios (i.e., when the number and/or length of loci is infinite), and yet, we currently have relatively little understanding of the empirical performance of the statistical binning pipeline itself when both the number and length of loci are bounded.

When considering the properties of the method, it is imperative to acknowledge that the statistical binning pipeline itself only infers a set of supergene alignments, not a species tree. Statistical binning is therefore *not* a species tree estimation method *per se*, it is a supergene estimation method that uses gene tree estimates to infer topology congruency among loci. Distinguishing between species tree estimation and supergene estimation is critical, because both are fundamentally different statistical problems: species tree estimation seeks a single species-

ACCEPTED MANUSCRIPT

level topology and set of parameters (i.e., divergence times, effective population sizes), while supergene inference involves deciding whether individual loci share the same gene tree or not. In this sense, statistical binning represents the first "cog in the wheel" of the phylogenomic analysis pipeline, which is followed by supergene tree estimation using standard phylogenetic techniques, such as maximum likelihood (ML) analysis, and species tree estimation using coalescent-based summary methods. Understanding whether the statistical binning pipeline provides reliable supergene alignments is therefore paramount to assessing the performance of the method. At the end of a statistical binning analysis, ML-analysis of each supergene is conducted under the assumptions of the standard phylogenetic model. While different supergenes can have different topologies, ML-analysis of the individual supergene alignments assumes that each gene placed within a supergene shares the same coalescent history. Under these conditions (i.e., a "true supergene" containing only congruent genes), standard ML-analysis – which assumes all sites share the same tree (Felsenstein, 1981) – will converge with increasing probability to the single, true gene tree as the length of each congruent locus in the supergene increase (Fig. 1, left).

In contrast, if a supergene incorrectly concatenates genes from multiple distinct topologies, standard ML-analysis of this "false supergene" will not converge to the true gene tree set (i.e., one tree for each distinct gene) as the length of each discordant gene increases, because it is restricted to inferring a single best-fit tree. In the right example shown in Figure 1, a false supergene has been constructed by concatenating three genes with conflicting genealogies (red, purple, green). Even if the length of each of the three genes is infinite, standard ML-analysis will infer only a single supergene tree – instead of the "true" gene tree set comprised of three distinct topologies. Violation of this fundamental assumption of the phylogenetic model (i.e., all sites share the same tree) is of major consequence because it is the underlying cause of the failure of

ACCEPTED MANUSCRIPT

ML-analysis in the presence of ILS (Mendes and Hahn, 2017), and can also cause other modeling pathologies and biases, such as SPILS ("substitutions produced by ILS"; Mendes and Hahn 2016). False supergene trees inferred using standard ML-analysis are likely to reflect an amalgamation of phylogenetic signal, such that the gene tree with the most support (i.e., highest number of informative sites) may have disproportionate influence. The overall supergene tree distribution will also likely be distorted as distinct gene trees are effectively "hidden" within false supergenes and may be poorly represented or absent in the set of supergene trees. False supergenes therefore represent profound phylogenetic model misspecification, and the hope is that methods such as statistical binning are able to avoid such sources of systematic bias by inferring accurate supergenes (i.e., Fig. 1 left vs. right).

A critical question therefore remains: how well does statistical binning infer topological congruency (or lack of) from gene tree estimates when attempting to construct true supergenes? Here we evaluate the performance of the method at this core function, and while previous studies have primarily focused on the theoretical properties of the method for species tree inference when aspects of the data are infinite (i.e., number of genes and/or gene lengths are unbounded), we take a decidedly different, model-based approach to understand whether statistical binning provides accurate supergenes or not. We conducted a post-hoc likelihood-based model assessment of statistical binning accuracy using the 14,446 alignments (8,251 exons, 2,516 introns, and 3,679 UCEs) and the corresponding set of 2,021 supergenes inferred for the original avian phylogenomic analyses (Jarvis et al., 2015, 2014). We specifically applied two different likelihood-based tests to characterize the accuracy of supergenes inferred via statistical binning: likelihood ratio tests (LRTs implemented in ConcatePillar; Leigh *et al.* 2008) and SH tests (Shimodaira and Hasegawa, 1999). The first approach conducts a series of likelihood-based

ACCEPTED MANUSCRIPT

model tests to evaluate whether the data (i.e., site patterns) of each respective supergene support a single topology or multiple, discordant topologies (Fig. 2, top box). The second method applies Shimodaira-Hasegawa tests (SH test; Shimodaira & Hasegawa 1999) to evaluate whether individual loci placed within a supergene reject the overall supergene tree in favor of a distinct, locus-specific topology (Fig. 2, bottom box). We used the results of the SH-tests to quantify the number of genes with evidence of significant topological congruency within each supergene alignment (i.e., genes that reject the supergene tree likely support a distinct topology). Unlike the statistical binning pipeline, which uses gene tree estimates to infer topological congruency, these two model-based approaches make direct use of the phylogenetic likelihood function by summing over site likelihoods for alternative tree models to validate supergene inferences by testing whether a single tree (i.e., "true positive", Fig. 1, left path) or multiple, distinct trees (i.e., "false positive") are a better explanation of the data (Fig. 1, right path).

2. Methods

2.1 Avian phylogenomic data

We downloaded the 14,446 alignments (8,251 exons, 2,516 introns, and 3,679 UCEs), the inferred supergene assignments for the 14,446 loci (i.e., assignment of each locus to a respective supergene), and the 2,021 ML supergene trees inferred via statistical binning for the avian phylogenomic analyses (Jarvis et al., 2015, 2014). For our simulation-based assessments of statistical binning accuracy, we downloaded the simulated gene tree sets and their associated inferred supergene assignments that were used in the original avian phylogenomic studies and were based on the estimated avian species tree (Jarvis et al., 2014; Mirarab et al., 2014).

ACCEPTED MANUSCRIPT

2.2 Likelihood-based tests of statistical binning accuracy

We evaluated the accuracy of each inferred supergene using likelihood ratio tests (LRTs) implemented in ConcatePillar (Leigh et al. 2008) and SH-tests (Shimodaira and Hasegawa, 1999) implemented in RAxML v8.0.0 (Stamatakis, 2014). First, we used Concatepillar to conduct LRTs to test whether a model consisting of a single topology or a model of multiple distinct topologies was better supported by the sequence data of each supergene based on the difference in log-likelihood scores between models (Fig. 2, top box). This approach effectively tests how many distinct topologies are supported by the data of each supergene and corrects for multiple comparisons throughout the process. If only a single topology best fits the data, this provides evidence that the supergene is likely to be accurate (i.e., Fig. 1, left). Conversely, if the data support multiple topologies, then the supergene likely violates the phylogenetic model because it exhibits evidence of incorrectly concatenated loci originating from distinct topologies (i.e., Fig. 1, right).

We used SH-tests in a similar fashion to test whether the difference in log-likelihood scores between the ML topology of each individual gene placed within a supergene and the overall ML supergene tree was statistically significant (Fig. 2, lower box). In other words, for each gene placed within an inferred supergene, we used SH-tests to compare the likelihood of the individual gene-specific ML topology with the overall supergene ML topology (Fig. 2, colored vs. gray trees in lower box). If the individual gene-specific ML tree was a statistically significant better fit than the supergene topology (i.e., $P < 0.05$), then that supergene was likely falsely constructed by statistical binning (i.e., concatenated loci with different phylogenetic histories, i.e., Fig. 1 right). The number of genes that reject the overall supergene tree in favor of a locus-specific tree provide an indication of the number of discordant genealogies present within a

ACCEPTED MANUSCRIPT

supergene alignment. SH-tests were conducted in RAxML 8.0.0 (Stamatakis, 2014) using the default GTR+I+**Γ** nucleotide substitution model independently for each locus.

In light of widespread evidence of supergene error (i.e., Fig 3), we were interested in characterizing the degree to which supergene trees reflected the topologies of their constituent genes. A critical concern of concatenating genes into a single supergene is that, if genes do not share the same tree, the gene with the most informative sites will dominate and overwhelm gene tree signals from shorter or less informative genes. In such cases, the supergene tree may only reflect the relationships supported by the dominant genealogy, while conflicting topologies of shorter loci will be effectively "hidden" and likely absent from the supergene tree distribution. To examine whether supergenes tend to be biased towards their longest constituent gene (and therefore capable of masking hidden gene trees from shorter gene constituents), we computed normalized Robinson-Foulds distance between each of the 14,446 gene trees and their associated supergene topology using the R package phangorn (Schliep, 2011).

2.3 Simulation-based assessment of statistical binning accuracy

We also evaluated the accuracy of statistical binning on the simulated gene tree sets provided in the original study (Jarvis et al., 2015; Mirarab et al., 2014), by testing whether supergenes inferred via the method included only simulated genes that share a common gene tree. For each inferred supergene, we computed pairwise Robinson-Foulds distances (Robinson and Foulds, 1979) between each simulated gene tree that statistical binning inferred to share a single supergene tree; all of the individual gene trees should be identical if statistical binning provided a correct supergene. An RF-distance of 0 between two trees means that the topologies are identical and an RF-distance >0 means the topologies are different. If all gene trees placed within a supergene have an RF-distance of 0, then the supergene was accurately inferred (i.e., Fig. 1, left).

ACCEPTED MANUSCRIPT

ACCEPTED MANUSCRIPT

If there is at least one RF-distance that is greater than 0, the supergene was inaccurate because it incorrectly concatenated loci that evolved along distinct, conflicting gene trees (i.e., Fig. 1, right). We computed unrooted RF-distances using the "multiRF" function provided in the phytools (Revell, 2012) package in R, and used these values to compute the mean RF-distance among gene trees across all inferred supergenes in each replicate simulation analysis (rightmost column of Supplementary Table 1). For reference, these supergenes were inferred in the original study using a bootstrap threshold of 75% (Jarvis et al., 2014).

2.4 Quantifying the impacts of statistical binning on gene tree distributions and species tree support

Considering evidence for spurious supergenes, we explored the impacts of statistical binning on both gene tree distributions and species tree support. To visualize differences in the underlying topological distributions due to statistical binning, we generated Densitree (Bouckaert, 2010) plots and summary consensus trees using TreeAnnotator (Rambaut and Drummond, 2016) of the unbinned gene tree and binned supergene tree distributions. We also quantified shifts in species tree support by measuring the difference in multispecies coalescent likelihoods of the unbinned gene trees and binned supergene trees using (1) the "unbinned" species tree (UST) estimated using the unbinned gene trees and (2) the "binned" species tree that was estimated using the binned supergene trees. For each of the 14,667 unbinned gene trees for the avian dataset, we measured the difference between the multispecies coalescent likelihood given the "binned" species tree and separately, the likelihood of the gene tree given the "unbinned" species tree: ΔGeneTreeLnL = LnL(GeneTree|Binned Species Tree) = LnL(GeneTree|Unbinned Species Tree). We also conducted this same analysis for the 2,021 supergenes inferred via statistical binning: ΔSupergeneTreeLnL = LnL(SupergeneTree|Binned Species Tree) =

ACCEPTED MANUSCRIPT

LnL(SupergeneTree|Unbinned Species Tree). To visualize the impacts of statistical binning on species tree support, we compared the distributions of the 14,667 ΔGeneTreeLnLs and the 2,021 ΔSupergeneTreeLnLs.

3. Results and Discussion

3.1 Evidence of widespread model misspecification due to statistical binning

Model-based evaluation of the performance of statistical binning on the avian phylogenomic data indicate that it does not provide reliable supergenes because it is highly prone to constructing "false supergenes" from loci with different coalescent histories – leading to profound and widespread phylogenetic model violation (Fig. 4). Both likelihood-based methods we employed indicate widespread error: 96.0% (1,940/2,021) and 92.3% (1,866/2,021) of supergenes concatenated multiple, conflicting topologies using the LRTs and SH-tests, respectively (Fig. 3a and 3b). Our results therefore indicate that the vast majority (>92%) of inferred supergenes represent false positives. We further evaluated the accuracy of statistical binning on the simulated datasets provided in the original avian study (Jarvis et al., 2015). Surprisingly, we found that 100% of multilocus supergenes (i.e., supergenes with at least 2 loci) across all simulation models and replicates were falsely constructed by statistical binning (Supplementary Table 1) and represent the right example shown in Figure 2. In other words, we found that the false positive rate of these methods for the avian dataset is ~92.3% at best.

Our analyses collectively suggest that statistical binning fails to overcome GTEE because it, like the methods it was designed to outperform, is based on unreliable gene tree and bootstrap support estimates that themselves suffer from high error, leading to false inferences of topological congruency. In other words, the core hypothesis test implemented in statistical

ACCEPTED MANUSCRIPT

binning, which uses bootstrap thresholds to determine gene tree congruence, does not appear to provide accurate supergene based upon our likelihood-based evaluations. Instead, our results indicate that genes incorrectly placed within these false supergenes exhibit surprisingly high gene tree incongruence, as indicated by mean Robinson-Foulds distances (RF-distance) within supergenes ranging from ~25-49 (Supplementary Table 1). ML-analysis of concatenated data predicts that supergene tree inference should be dominated by the gene with the most informative sites, which was observed in our analysis (Fig. 4). Considering our evidence of widespread supergene error (Fig. 3), evidence of the dominance of the longest gene driving supergene tree estimates suggests that alternative topologies of other, shorter genes within supergenes are likely under-represented or even absent from false supergene trees. At best, this scenario would result in the massive loss of genealogical information due to binning genes into supergenes (i.e., only the topology from the longest gene is represented). A potential and worse scenario would be that this amalgamation of signal from genes with different genealogies may instead led to totally spurious supergene estimates that do not overlap with any of the true gene trees underlying the data (i.e., unnatural products of signal averaging). These findings also further clarify the underlying reason for the reported distortion of supergene tree distributions resulting from statistical binning (Liu and Edwards, 2015), and corroborate recent theoretical work that has shown the inconsistency of statistical binning when the length of each locus is finite (Roch et al., 2018).

To characterize the impacts of statistical binning and potential biases it introduces in gene tree distributions, we compared the distribution of supergene trees with the distribution of locus-specific gene trees. Overlays of gene trees using Densitree illustrate that binning leads to major shifts in the gene tree distributions, including several major decreases in conflict (and increases

ACCEPTED MANUSCRIPT

ACCEPTED MANUSCRIPT

in gene tree resolution), particularly for more ancient nodes (Figs. 5a-b), consistent with previous evidence that binning ‘flattens’ gene tree distributions (Liu et al., 2015a). Similarly, comparison of consensus trees between binned and unbinned gene tree sets highlight major differences in gene tree topology and broad increases in bipartition agreement based on binned supergene trees (Figs. 5c-d). Comparisons of likelihood support for alternative species trees indicates that statistical binning introduces major changes in the shape and magnitude of variation of species tree likelihoods (Figs. 5e-f). For example, the number of supergene trees that strongly support one species tree over another increases compared to the unbinned gene trees. Considering evidence that a large proportion of supergenes may be false (e.g., Fig. 3), our results collectively suggest that statistical binning strongly biases gene tree distributions that do not reflect the true gene tree variation, and thereby provide high support for an incorrect species tree.

Although we have primarily presented the problem of “false supergenes” as a dichotomous phenomenon (i.e., ether all genes are congruent or not), their impacts on species tree estimation may be more complex depending on the particular evolutionary parameters (i.e., species tree shape, divergence times, population sizes), and/or experimental conditions (i.e., number and length of loci). For example, “false supergenes” comprised of only two distinct trees may be less problematic then if they contain loci from three distinct trees. It also seems possible that particular branches and subclades may be more or less accurately estimated than others. This could occur, for example, if most genes within a false supergene agree on the placement of a particular clade. Deeper nodes may be more accurately estimated than more recent species splits – perhaps because individual genes may exhibit little conflict in the placement of more ancient lineages (i.e., most ancient lineages are completely sorted). Nonetheless, ML-analysis of false supergenes will be a forced comprise of the conflicting signal exhibited across incongruent loci

ACCEPTED MANUSCRIPT

and thus, will likely suffer large-scale systematic error in topology, branch length estimates, and other parameters.

4. Conclusions

Perhaps surprisingly, genome-scale datasets do not yet equate to straight-forward and robust resolution of phylogeny. Instead, both biology and methodology continue to pose serious challenges for phylogenomic analyses. There is certainly logical merit in approaches that are designed – at least in theory – to tractably address these issues, such as statistical binning. Our results, however, suggest that nearly every supergene tree inferred via this approach and used to reconstruct the avian species tree is likely to suffer extensive systematic error at the hands of pervasive phylogenetic model misspecification, such that statistical binning is more likely to suffer the effects of GTEE and ILS than overcome them. Instead, the effects of ILS will be rampant in the set of ML supergenes trees used to estimate a species tree when statistical binning is applied. Because these methods only infer topological congruency and do not estimate a species tree, we also argue that model-based supergene validation of statistical binning inferences (i.e., LRT tests) provides a far more direct assessment of the method at its core function and brings clarity to previous arguments, which primarily evaluated the performance of downstream species tree estimation methods that use supergenes as input data.

These findings raise the question of what alternative strategies would be useful for avoiding these issues? One solution is to simply collect more genetically linked data per locus (i.e., longer orthologus loci) to obtain higher quality gene trees without the need for concatenation. In practice, however, “simply collecting more data” is not always a simple or even viable option, particularly given that the original avian analyses sampled whole-genomes and still faced these

ACCEPTED MANUSCRIPT

issues, in part due to the difficulties in aligning long orthologous regions across deep evolutionary time. Increasing the length of individual loci also has the downside of increasing the probability of intra-locus recombination, which may pose additional complications and violations of the phylogenetic model analogous to those introduced by erroneous supergenes. Indeed, false supergenes exemplify the most "extreme" form of this violation whereby recombination occurs freely between genes with non-congruent histories incorrectly placed within a supergene. Unlike binning approaches that "agnostically" infer supergenes using only gene tree estimates without taking into account genome structure, it may prove fruitful to make effective use of known genetic linkage among loci to propose the combinability of nearby putatively linked loci and test this inference using model-based approaches. Above all, our findings highlight the critical need for the continued development of more accurate phylogenomic methods that can tractably and reliably deliver more reliable gene trees, and ultimately, better species tree estimates

Acknowledgments

This project was supported by an NSF grant (DEB-1655571) to TAC, computation resources provided by the Texas Advanced Computing Center (TACC), and a University of Texas at Arlington Phi Sigma Society grant to RHA.

References

Bayzid, M.S., Mirarab, S., Boussau, B., Warnow, T., 2015. Weighted Statistical Binning: Enabling Statistically Consistent Genome-Scale Phylogenetic Analyses. PLoS One 10,

ACCEPTED MANUSCRIPT

ACCEPTED MANUSCRIPT

e0129183. https://doi.org/10.1371/journal.pone.0129183

Blaimer, B.B., LaPolla, J.S., Branstetter, M.G., Lloyd, M.W., Brady, S.G., 2016. Phylogenomics, biogeography and diversification of obligate mealybug-tending ants in the genus Acropyga. Mol. Phylogenet. Evol. 102, 20–29. https://doi.org/10.1016/j.ympev.2016.05.030

Bouckaert, R.R., 2010. DensiTree: Making sense of sets of phylogenetic trees. Bioinformatics. https://doi.org/10.1093/bioinformatics/btq110

Branstetter, M.G., Danforth, B.N., Pitts, J.P., Faircloth, B.C., Ward, P.S., Buffington, M.L., Gates, M.W., Kula, R.R., Brady, S.G., 2017. Phylogenomic Insights into the Evolution of Stinging Wasps and the Origins of Ants and Bees. Curr. Biol. 27, 1019–1025. https://doi.org/10.1016/j.cub.2017.03.027

Degnan, J.H., Rosenberg, N.A., 2009. Gene tree discordance, phylogenetic inference and the multispecies coalescent. Trends Ecol. Evol. https://doi.org/10.1016/j.tree.2009.01.009

Edwards, S. V, Liu, L., Pearl, D.K., 2007. High-resolution species trees without concatenation. Proc. Natl. Acad. Sci. U. S. A. 104, 5936–5941. https://doi.org/10.1073/pnas.0607004104

Felsenstein, J., 1981. Evolutionary trees from DNA sequences: A maximum likelihood approach. J. Mol. Evol. 17, 368–376. https://doi.org/10.1007/BF01734359

Heled, J., Drummond, A.J., 2010. Bayesian Inference of Species Trees from Multilocus Data. Mol. Biol. Evol. 27, 570–580. https://doi.org/10.1093/molbev/msp274

Jarvis, E.D., Mirarab, S., Aberer, A.J., Li, B., Houde, P., Li, C., Al., E., 2014. Whole-genome analyses resolve early branches in the tree of life of modern birds. Science (80-.). 346, 1320–1331. https://doi.org/10.1126/science.1253451

Jarvis, E.D., Mirarab, S., Aberer, A.J., Li, B., Houde, P., Li, C., Ho, S.Y.W., Faircloth, B.C., Nabholz, B., Howard, J.T., Suh, A., Weber, C.C., da Fonseca, R.R., Alfaro-Núñez, A., Narula, N., Liu, L., Burt, D., Ellegren, H., Edwards, S. V, Stamatakis, A., Mindell, D.P., Cracraft, J., Braun, E.L., Warnow, T., Jun, W., Gilbert, M.T.P., Zhang, G., 2015. Phylogenomic analyses data of the avian phylogenomics project. Gigascience 4, 4. https://doi.org/10.1186/s13742-014-0038-1

JeŠovnik, A., Sosa-Calvo, J., Lloyd, M.W., Branstetter, M.G., FernÁndez, F., Schultz, T.R., 2017. Phylogenomic species delimitation and host-symbiont coevolution in the fungus-farming ant genus Sericomyrmex Mayr (Hymenoptera: Formicidae): ultraconserved elements (UCEs) resolve a recent radiation. Syst. Entomol. 42, 523–542. https://doi.org/10.1111/syen.12228

Knowles, L.L., 2009. Estimating species trees: methods of phylogenetic analysis when there is incongruence across genes. Syst. Biol. 58, 463–467.

Kubatko, L.S., Degnan, J.H., 2007. Inconsistency of phylogenetic estimates from concatenated data under coalescence. Syst. Biol. 56, 17–24. https://doi.org/10.1080/10635150601146041

Leigh, J.W., Susko, E., Baumgartner, M., Roger, A.J., 2008. Testing congruence in

ACCEPTED MANUSCRIPT

ACCEPTED MANUSCRIPT

phylogenomic analysis. Syst. Biol. 57, 104–115. https://doi.org/10.1080/10635150801910436

Leigh, J.W., Susko, E., Baumgartner, M., Roger, A.J., 2008. Testing congruence in phylogenomic analysis. Syst. Biol. 57, 104–115. https://doi.org/10.1080/10635150801910436

Liu, L., 2008. BEST: Bayesian estimation of species trees under the coalescent model. Bioinformatics 24, 2542–2543. https://doi.org/10.1093/bioinformatics/btn484

Liu, L., Edwards, S. V, 2015. Comment on "Statistical binning enables an accurate coalescent-based estimation of the avian tree." Science (80-.). 350, 171.

Liu, L., Edwards, S. V, Mirarab, S., Bayzid, M.S., Boussau, B., Warnow, T., 2015a. Comment on "Statistical binning enables an accurate coalescent-based estimation of the avian tree." Science (80-.). 350, 171 LP-171.

Liu, L., Wu, S., Yu, L., 2015b. Coalescent methods for estimating species trees from phylogenomic data. J. Syst. Evol. https://doi.org/10.1111/jse.12160

Liu, L., Yu, L., Edwards, S. V, 2010. A maximum pseudo-likelihood approach for estimating species trees under the coalescent model. BMC Evol. Biol. 10, 302. https://doi.org/10.1186/1471-2148-10-302

Malmstrøm, M., Matschiner, M., Tørresen, O.K., Star, B., Snipen, L.G., Hansen, T.F., Baalsrud, H.T., Nederbragt, A.J., Hanel, R., Salzburger, W., Stenseth, N.C., Jakobsen, K.S., Jentoft, S., 2016. Evolution of the immune system influences speciation rates in teleost fishes. Nat. Genet. 48, 1204–1210. https://doi.org/10.1038/ng.3645

Mendes, F.K., Hahn, M.W., 2017. Why Concatenation Fails Near the Anomaly Zone. Syst. Biol. https://doi.org/10.1093/sysbio/syx063

Mendes, F.K., Hahn, M.W., 2016. Gene tree discordance causes apparent substitution rate variation. Syst. Biol. 65, 711–721. https://doi.org/10.1093/sysbio/syw018

Mirarab, S., Bayzid, M.S., Boussau, B., Warnow, T., 2015. Response to Comment on "Statistical binning enables an accurate coalescent-based estimation of the avian tree." Science (80-.). 350, 171–171. https://doi.org/10.1126/science.aaa7719

Mirarab, S., Bayzid, S.M., Boussau, B., Warnow, T., 2014. Statistical binning enables an accurate coalescent-based estimation of the avian tree. Science (80-.). 346, 1250463–1250463. https://doi.org/10.1126/science.1250463

Platt, R.N., Faircloth, B.C., Sullivan, K.A.M., Kieran, T.J., Glenn, T.C., Vandewege, M.W., Lee, T.E., Baker, R.J., Stevens, R.D., Ray, D.A., 2018. Conflicting Evolutionary Histories of the Mitochondrial and Nuclear Genomes in New World Myotis Bats. Syst. Biol. 67, 236–249. https://doi.org/10.1093/sysbio/syx070

Prum, R.O., Berv, J.S., Dornburg, A., Field, D.J., Townsend, J.P., Lemmon, E.M., Lemmon, A.R., 2015. A comprehensive phylogeny of birds (Aves) using targeted next-generation

ACCEPTED MANUSCRIPT

ACCEPTED MANUSCRIPT

DNA sequencing. Nature 526, 569–573. https://doi.org/10.1038/nature15697

Rambaut, A., Drummond, A.J., 2016. TreeAnnotator v1.8.4 [WWW Document]. http://beast.bio.ed.ac.uk/. https://doi.org/Doi 10.1246/Bcsj.82.1052

Revell, L.J., 2012. phytools: An R package for phylogenetic comparative biology (and other things). Methods Ecol. Evol. 3, 217–223. https://doi.org/10.1111/j.2041-210X.2011.00169.x

Robinson, D., Foulds, L., 1979. Comparison of weighted labelled trees. Lect. Notes Math. 748, 119–126. https://doi.org/10.1007/BFb0102678

Roch, S., Nute, M., Warnow, T., 2018. Long-branch attraction in species tree estimation: inconsistency of partitioned likelihood and topology-based summary methods. arXiv Prepr. arXiv1803.02800.

Roch, S., Warnow, T., 2015. On the Robustness to Gene Tree Estimation Error (or lack thereof) of Coalescent-Based Species Tree Methods. Syst. Biol. 64, 663–676. https://doi.org/10.1093/sysbio/syv016

Schliep, K.P., 2011. phangorn: Phylogenetic analysis in R. Bioinformatics. https://doi.org/10.1093/bioinformatics/btq706

Shimodaira, H., Hasegawa, M., 1999. Multiple Comparisons of Log-Likelihoods with Applications to Phylogenetic Inference. Mol. Biol. Evol. 16, 1114–1116. https://doi.org/10.1093/oxfordjournals.molbev.a026201

Stamatakis, A., 2014. RAxML version 8: A tool for phylogenetic analysis and post-analysis of large phylogenies. Bioinformatics 30, 1312–1313. https://doi.org/10.1093/bioinformatics/btu033

Streicher, J.W., Miller, E.C., Guerrero, P.C., Correa, C., Ortiz, J.C., Crawford, A.J., Pie, M.R., Wiens, J.J., 2018. Evaluating methods for phylogenomic analyses, and a new phylogeny for a major frog clade (Hyloidea) based on 2214 loci. Mol. Phylogenet. Evol. 119, 128–143.

Tarver, J.E., Dos Reis, M., Mirarab, S., Moran, R.J., Parker, S., O'Reilly, J.E., King, B.L., O'Connell, M.J., Asher, R.J., Warnow, T., Peterson, K.J., Donoghue, P.C.J., Pisani, D., 2016. The interrelationships of placental mammals and the limits of phylogenetic inference. Genome Biol. Evol. 8, 330–344. https://doi.org/10.1093/gbe/evv261

Warnow, T., 2015. Concatenation analyses in the presence of incomplete lineage sorting. PLoS Curr. 7. https://doi.org/10.1371/currents.tol.8d41ac0f13d1abedf4c4a59f5d17b1f7

ACCEPTED MANUSCRIPT

ACCEPTED MANUSCRIPT

Figure Legends

FIGURE 1. Statistical binning is a supergene estimation method, not a species tree estimation method. Based on similarities (or lack of) among gene tree estimates and bootstrap support values, the core function of the method is to infer whether individual genes share a common genealogy, and if so, concatenate congruent genes to construct longer supergenes. Example indicating loci sampled from two different chromosomes and three distinct gene trees (red, blue, and purple). If statistical binning is accurate, inferred supergenes will only concatenate loci that share the same topology (i.e., left example showing a "true supergene" comprised entirely of red loci). ML-analyses of "true supergenes" (MLE indicated as gray tree) will converge to the true topology as the length of each congruent locus increases, because all sites in the alignment evolved under the same red topology. However, if statistical binning is not accurate, incongruent loci that do not share a common topology may be incorrectly concatenated to form "false supergenes". In the right example, a false supergene has been constructed from three genes with three different topologies (blue, red, and purple). False supergenes represent profound phylogenetic model misspecification, because standard ML-analysis assumes that all sites within an alignment evolved under the same tree, and thus, only one tree will be estimated when there should be three (right example). Regardless of whether this ML topology is the blue, red, purple, or some other topology, the answer is the same: ML-analysis cannot be statistically consistent because it cannot estimate three unique trees. False supergene trees are likely to reflect an amalgamation of conflicting phylogenetic signal (here three distinct trees), such that the gene tree with the most support (i.e., highest number of informative sites) may have disproportionate influence (see Fig. 4). The relevant questions is thus whether statistical binning tends to infer true supergenes (left) or false supergenes (right), and although the method does not directly

ACCEPTED MANUSCRIPT

ACCEPTED MANUSCRIPT

estimate a species tree, clearly supergene accuracy is likely to influence downstream species tree accuracy.

FIGURE 2. Model-based assessment of statistical binning accuracy. We tested the accuracy of each of the 2,021 supergenes inferred from the Avian phylogenomics project using Likelihood Ratio Tests (LRTs, implemented in Concatepillar, top box) and Shimodaira-Hasegawa (SH-tests, bottom box). The LRTs approach tests how many distinct topologies are present in a supergene inferred via statistical binning. For example, the likelihood of a model consisting of three distinct trees (red, blue and purple in top box) is compared to single-tree model (gray alignment and tree in top box). Similarly, the SH-tests approach evaluates whether individual loci placed within a supergene reject the overall supergene topology in favor of a locus-specific topology (i.e., red vs. gray supergene topology shown in lower box). A "true supergene" and its associated supergene tree are considered accurate if only a single topology is supported by the data (i.e., Fig. 1 left), while a "false supergene" occurs when multiple trees are supported by the data (i.e., Fig. 1 right). For both methods, we quantified the number and fraction of "true supergenes" (blue bar in right histogram) and "false supergenes" that incorrectly concatenate multiple trees (2-8 in this case, black bars and red area).

FIGURE 3. More than 92% of supergenes inferred via statistical binning appear to be false positives. Histograms showing the number of distinct topologies inferred with (a) likelihood ratio tests (LRTs with Concatepillar) and (b) Shimodaira-Hasegawa (SH-tests) across the 2,021 supergenes inferred for the Avian phylogenomic analyses. LRTs (a) and SH-tests (b) indicate that over 96% (1,934/2,021) and 92% (1,866/2,021) of supergenes are false positives, respectively (black bars and red area). In other words, only 4% (81/2,021) of supergenes appear

ACCEPTED MANUSCRIPT

to be “true supergenes” based on LRTs (blue bar), and only 7.7% (155/2, 021) based on SH-tests (blue bar).

FIGURE 4. Robinson-Foulds (RF) distances between an individual gene topology and its associated supergene topology decrease with relative gene length, such that supergenes inferred via statistical binning tend to be biased towards the topology of its longest gene. Boxplots indicate the distribution of RF distances between each gene-specific ML topology and its respective supergene ML topology ranked from shortest to longest relative gene length. Results shown for supergenes comprised of 7 genes (a) and 8 genes (b), respectively.

FIGURE 5. The impacts of statistical binning on gene tree distributions and species tree support. Densitree plots showing the gene tree topology distribution for (a) the individual gene trees (“unbinned”) and (b) the supergene trees. Plot of consensus trees with bipartition frequencies estimated using the individual, unbinned gene trees (c) and (d) the supergene trees constructed with statistical binning (d). Node circles are labeled and colored by the bipartition frequencies observed in their respective gene tree distributions. Histograms showing the distributions of multispecies coalescent likelihoods for the unbinned gene trees (ΔGeneTreeLnLs; e) and binned supergene trees (ΔSupergeneTreeLnLs; f).

ACCEPTED MANUSCRIPT

ACCEPTED MANUSCRIPT

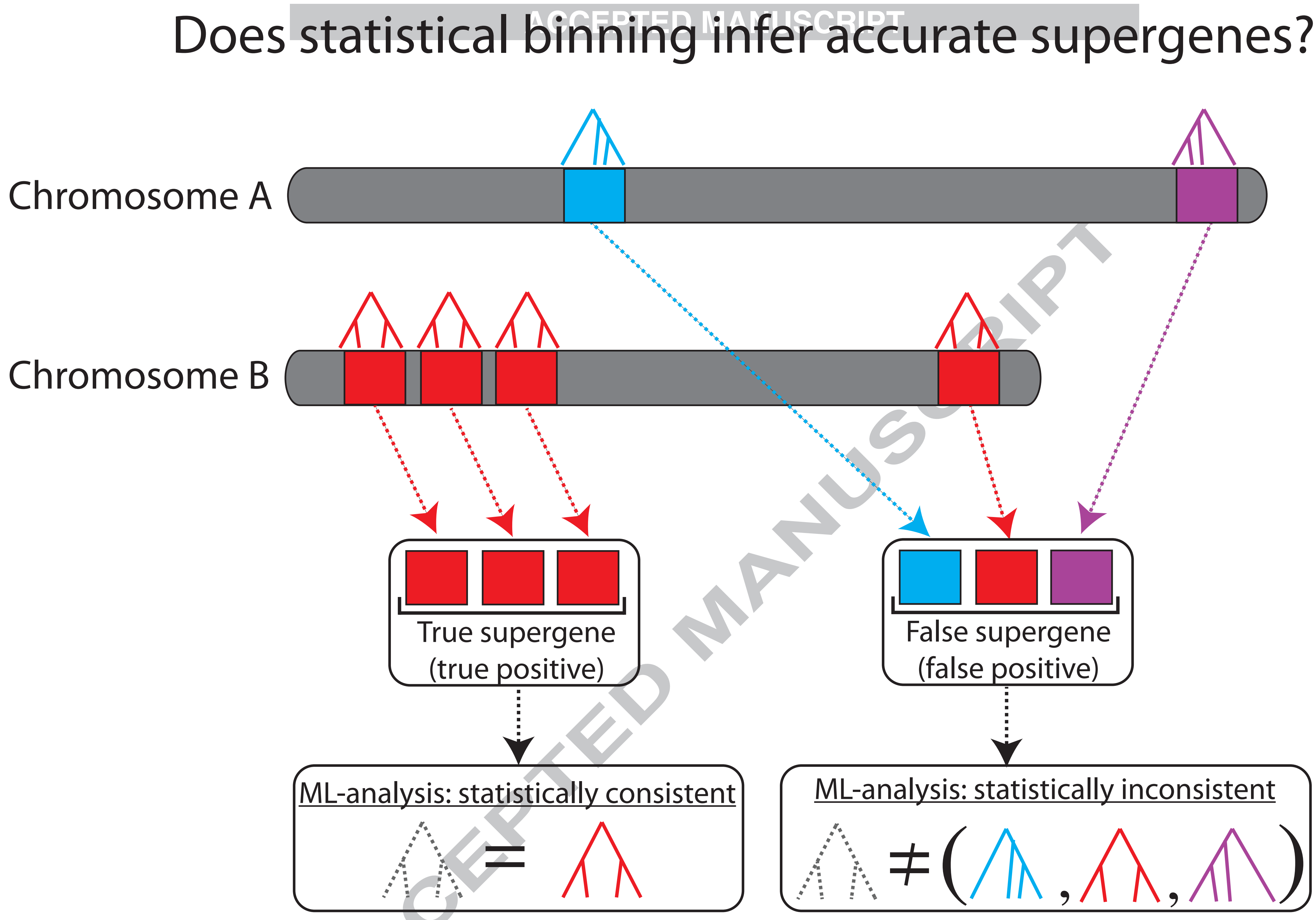

ACCEPTED MANUSCRIPT

ACCEPTED MANUSCRIPT

Highlights

- We conduct model-based evaluation of statistical binning accuracy using empirical and simulated data from the avian phylogenomics analyses
- Our results indicate pervasive and profound phylogenetic model misspecification (>90% false positives) with the use of binning techniques
- The vast majority of supergenes inferred via this method represent "false supergenes" that incorrectly concatenate loci with conflicting coalescent histories
- We find evidence that trees reconstructed from false supergenes mask hidden genealogies and thus, may bias downstream species inferences

ACCEPTED MANUSCRIPT

ACCEPTED MANUSCRIPT

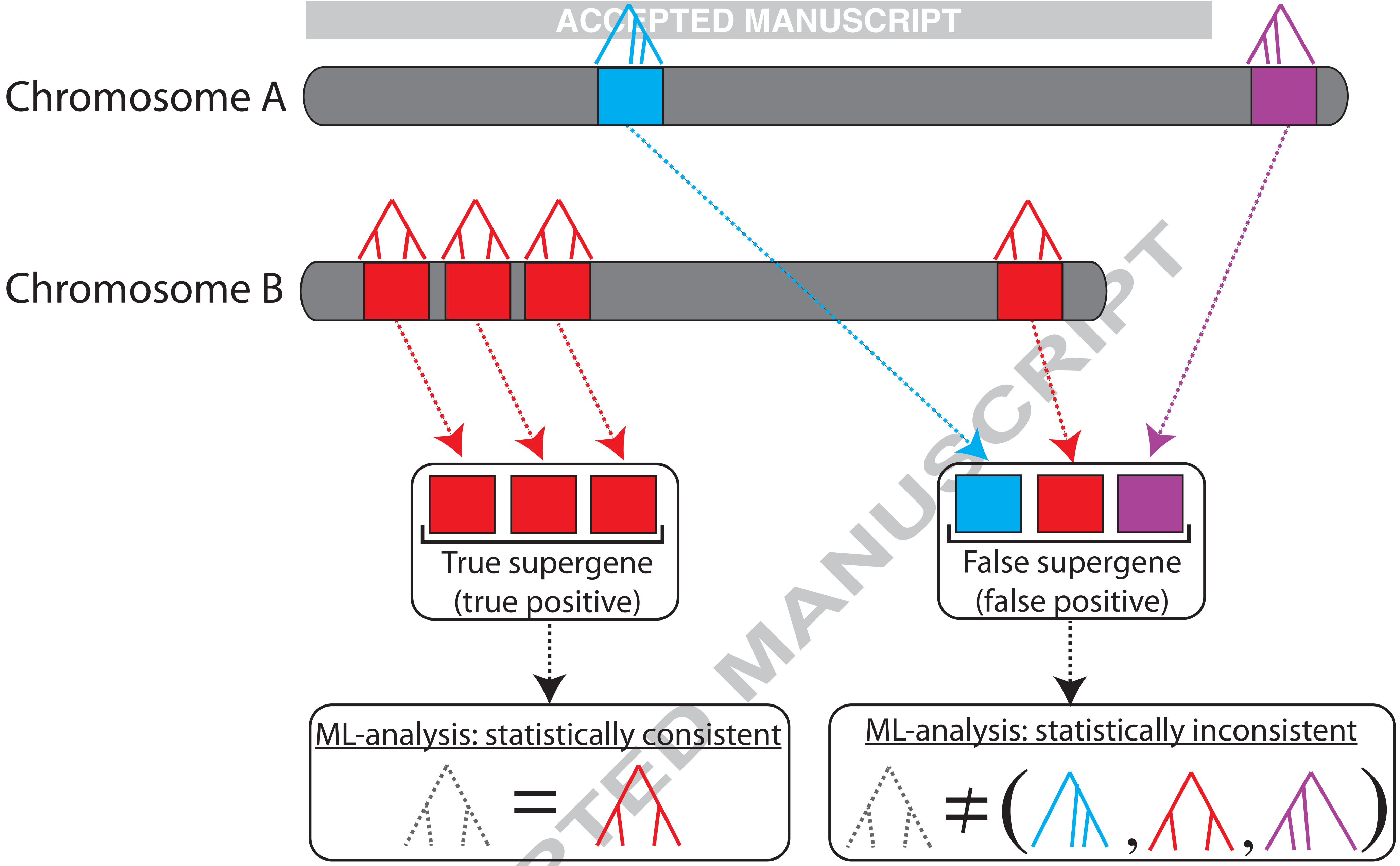

ACCEPTED MANUSCRIPT

ACCEPTED MANUSCRIPT

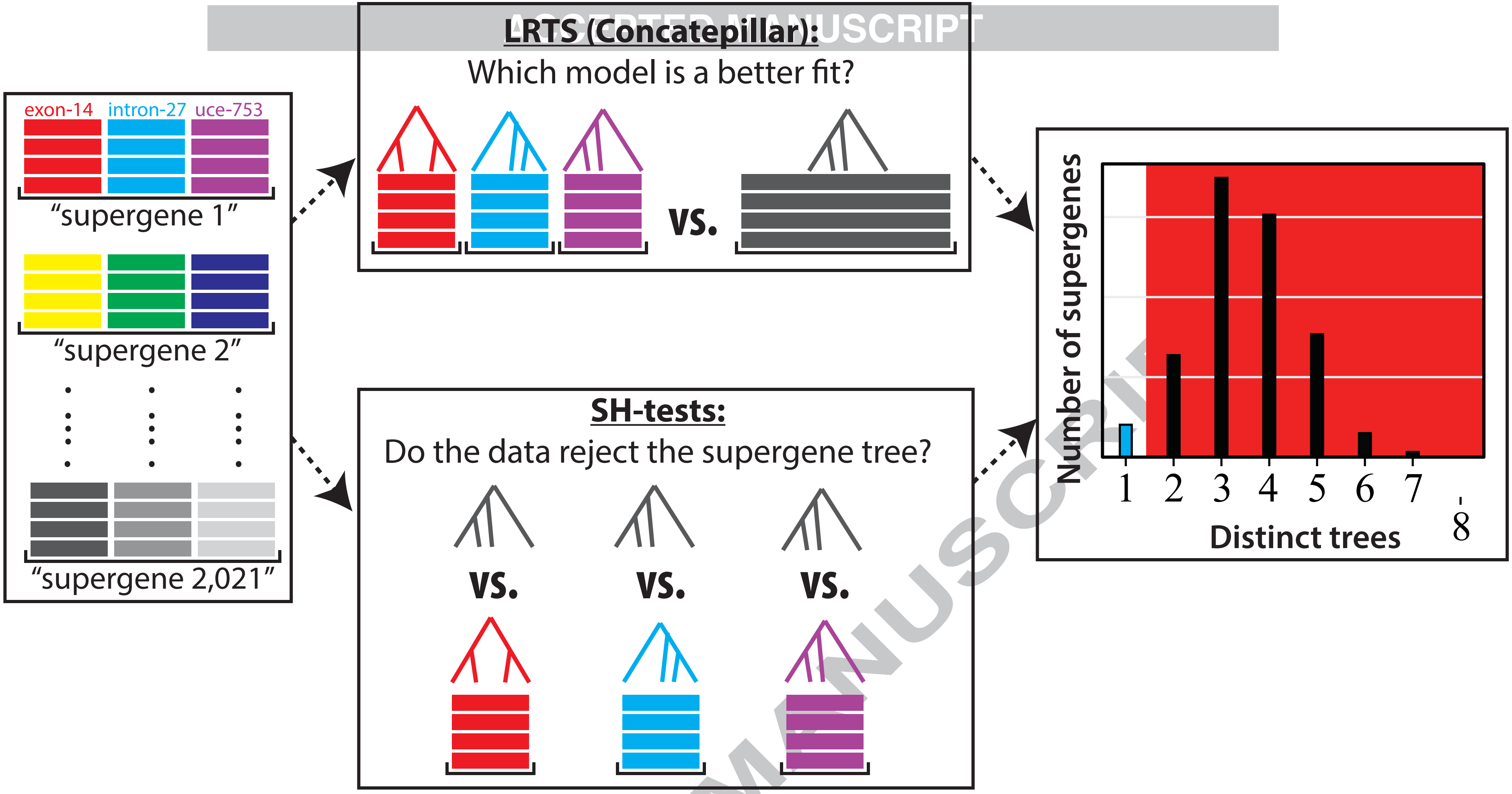

ACCEPTED MANUSCRIPT

A)

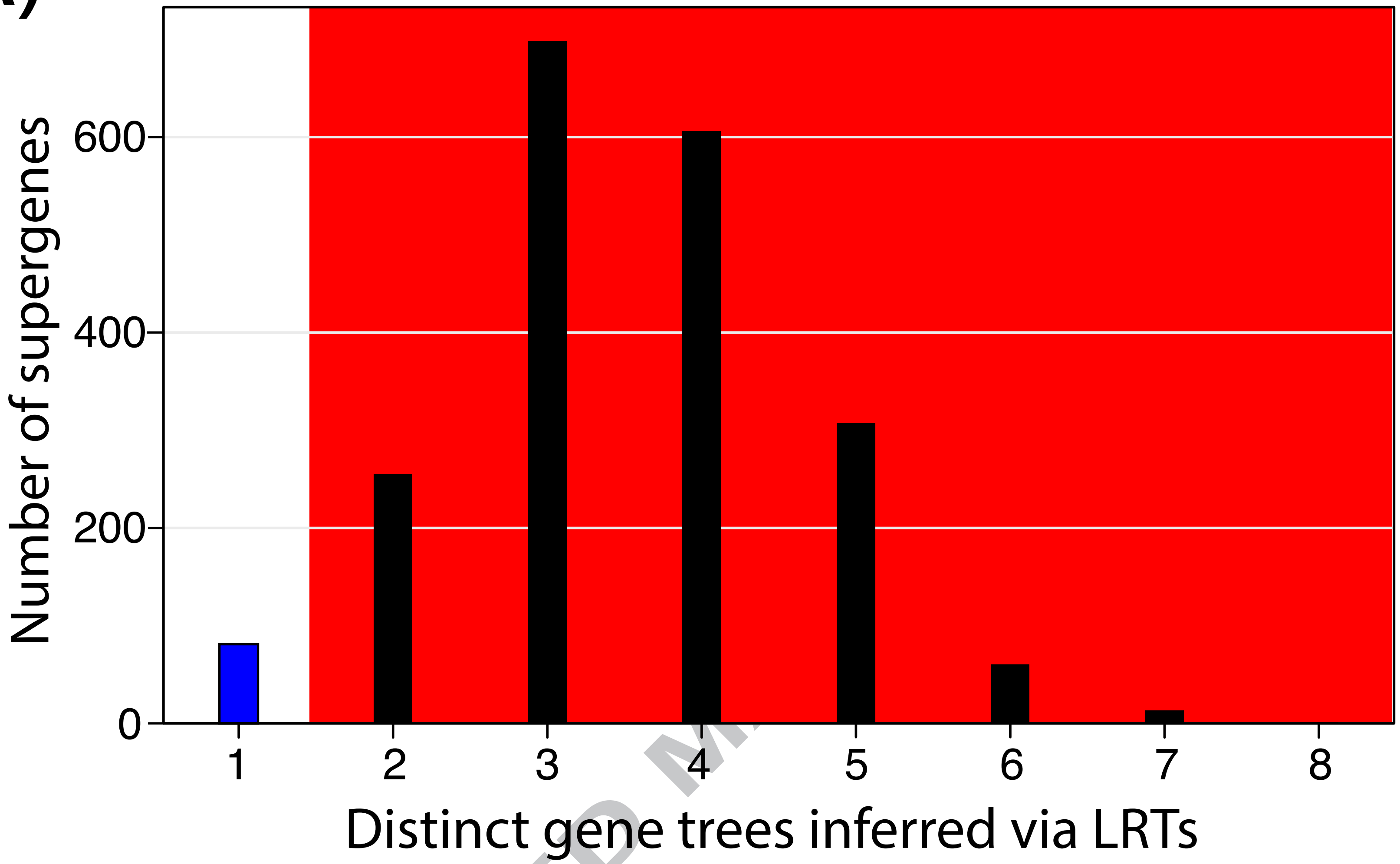

B)

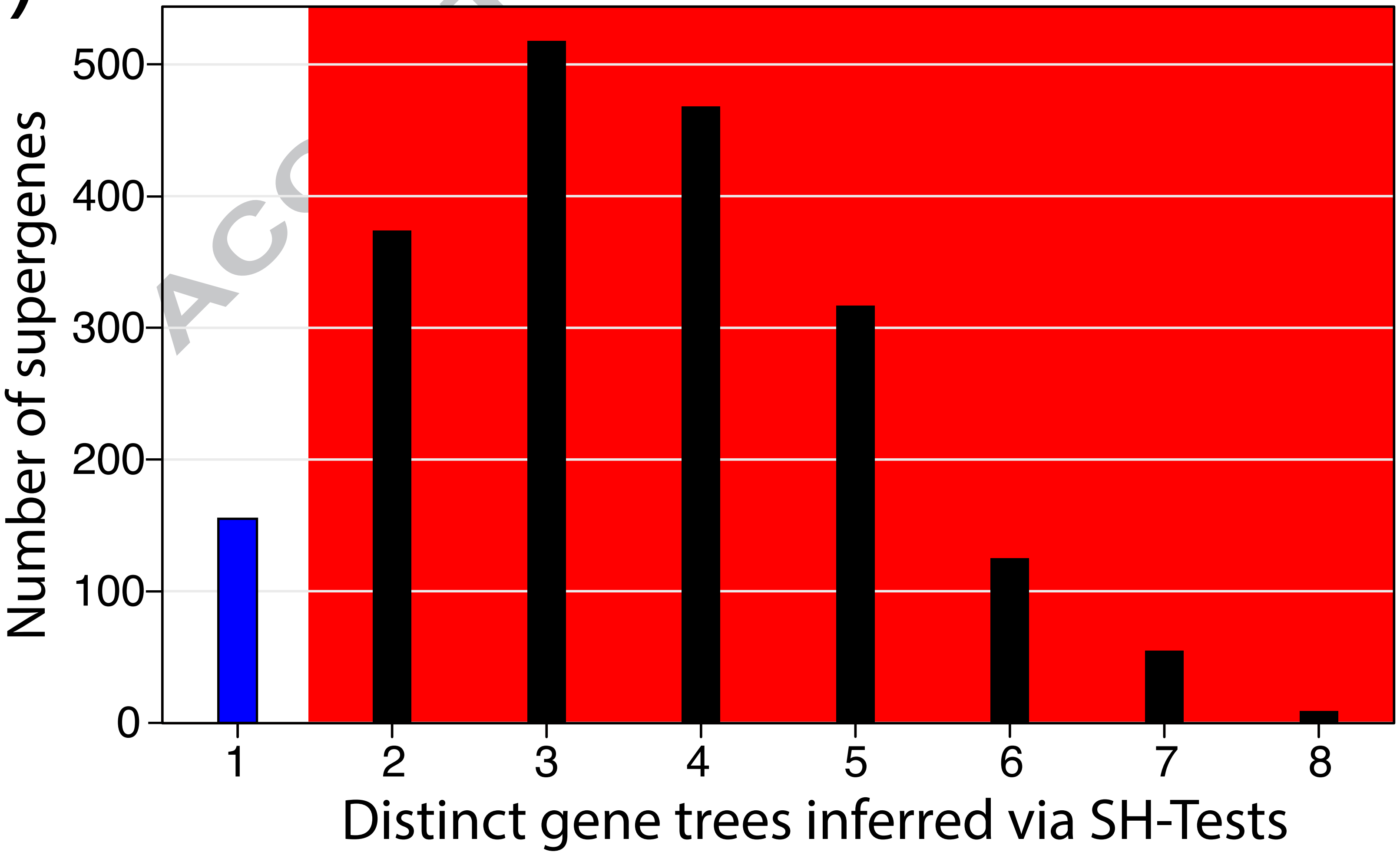

ACCEPTED MANUSCRIPT

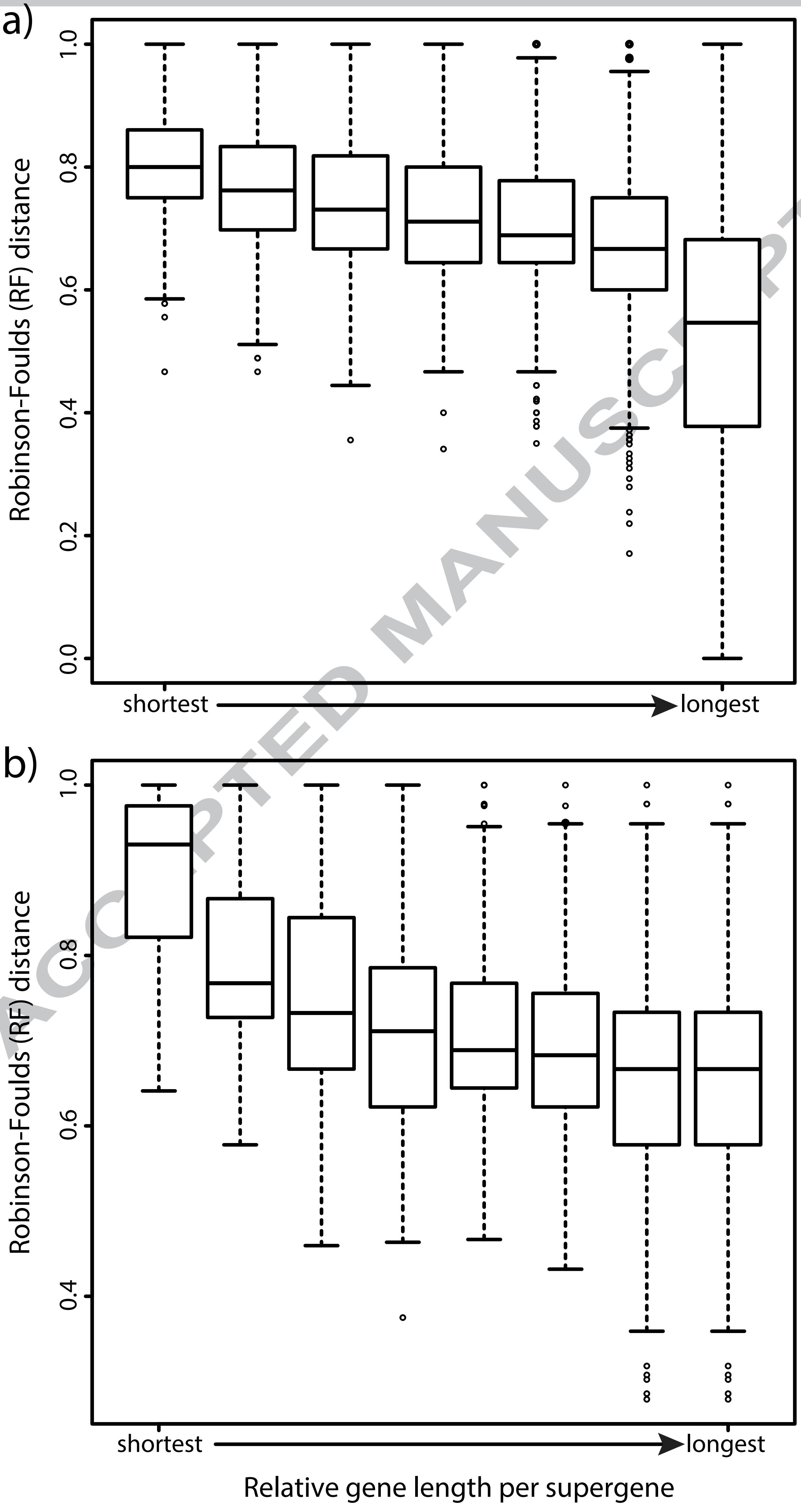

ACCEPTED MANUSCRIPT

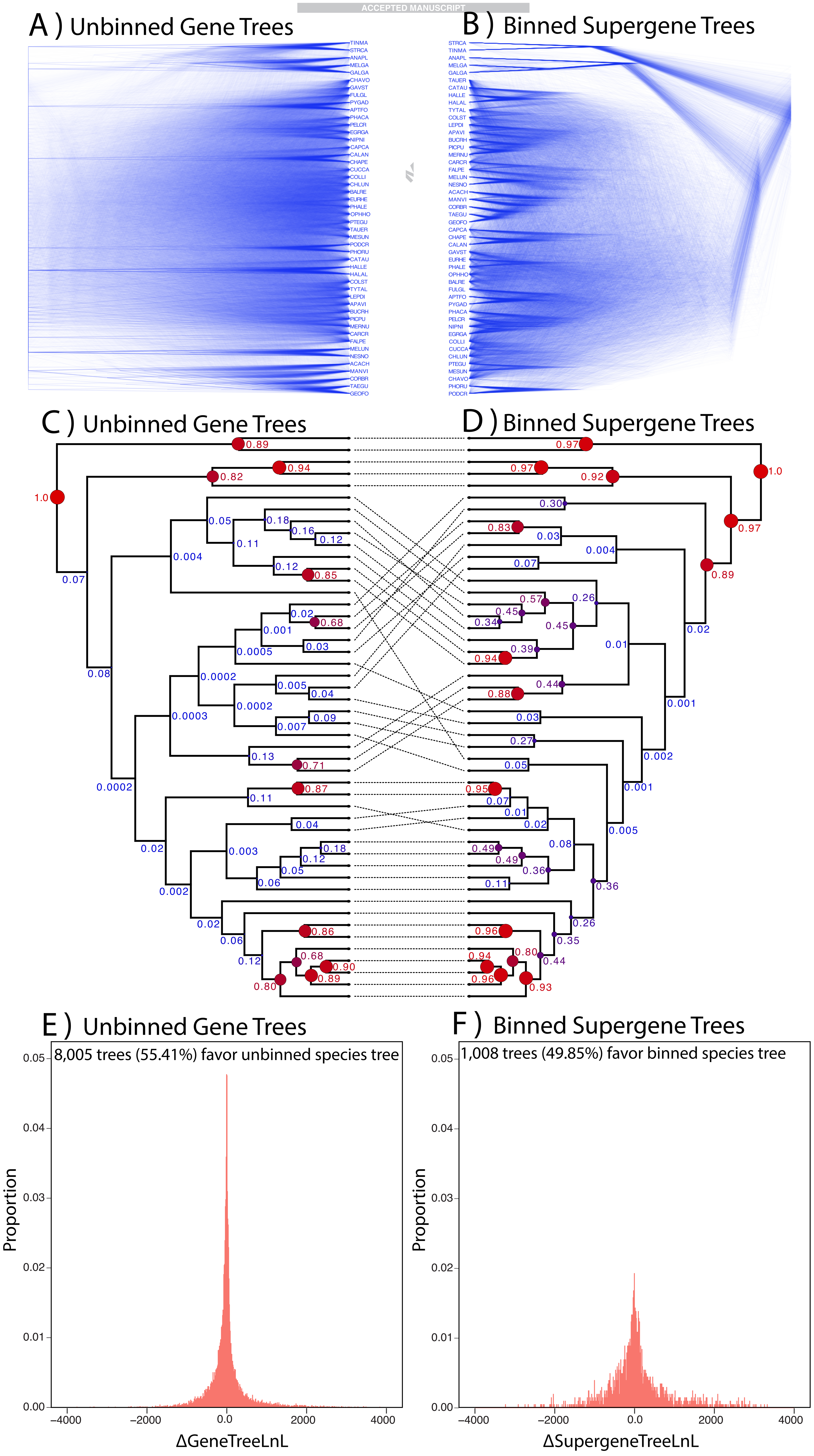

Supplement: Supplementary file 1 [file mmc1.pdf]
